# From Envelope to Encephalopathy: How HIV-1 gp120 Drives Neurocognitive Decline

**DOI:** 10.3390/v18050495

**Published:** 2026-04-24

**Authors:** Maryline Santerre, Jenny Shrestha, Charles N. S. Allen, Natalia Shcherbik, Bassel E. Sawaya

**Affiliations:** 1 FELS Cancer Institute for Personalized Medicine, Lewis Katz School of Medicine, Temple University, 3307 North Broad Street, Philadelphia, PA 19140, USA; marylinesanterre@gmail.com (M.S.); jenny.shrestha@bms.com (J.S.); charlesnallen85@gmail.com (C.N.S.A.); 2 Department of Cell and Molecular Biology, School of Osteopathic Medicine, Rowan University, 2 Medical Center Drive, Stratford, NJ 08084, USA; shcherna@rowan.edu; 3 Department of Cancer and Cellular Biology, Lewis Katz School of Medicine, Temple University, Philadelphia, PA 19140, USA; 4 Department of Neural Sciences, Lewis Katz School of Medicine, Temple University, Philadelphia, PA 19140, USA

**Keywords:** HIV-1 gp120, neurotoxicity, mitochondrial dysfunction, NMDA receptor, BDNF, CREB, oxidative stress, neuroprotection, extracellular vesicles, senescence, calcium signaling

## Abstract

Although neurons are not productively infected by HIV-1, the envelope glycoprotein gp120, detectable in cerebrospinal fluid independently of active viral replication, gains intraneuronal access via lipid raft-mediated endocytosis, macropinocytosis, and retrograde axonal transport, contributing to persistent neurobiological dysfunction within the central nervous system. Once internalized, gp120 is associated with neuronal dysfunction involving convergent pathways, including excitotoxic calcium dysregulation, mitochondrial and metabolic failure, and inflammatory and senescence-associated amplification. These pathways converge on suppression of CREB and BDNF signaling, dismantling the transcriptional and neurotrophic programs required for synaptic maintenance and cognitive resilience. Extracellular vesicle-mediated dissemination and microRNA reprogramming extend gp120-associated neurobiological effects beyond sites of receptor engagement, while gut-derived metabolites, particularly quinolinic acid, lower the excitotoxic threshold through synergistic activation of NMDA receptors. Together, these mechanisms define HAND as a network disorder in which gp120 contributes to persistent neurocognitive dysfunction beyond active viral replication, identifying convergent therapeutic nodes where combination strategies targeting excitotoxicity, mitochondrial dysfunction, and neuroinflammation offer the most promising path toward durable neuroprotection.

## 1. Introduction

### 1.1. HIV-Associated Neurocognitive Disorders in the Modern Era

The introduction of combination antiretroviral therapy (cART) transformed HIV-1 infection into a manageable chronic condition, dramatically extending life expectancy [1]. However, this success has revealed a persistent challenge: aging HIV-infected individuals experience accelerated biological aging and increased prevalence of neurocognitive impairment that cART alone cannot fully prevent [2]. HIV-associated neurocognitive disorders (HAND) encompass a spectrum from asymptomatic neurocognitive impairment through mild neurocognitive disorder to HIV-associated dementia [3], with milder forms persisting in a substantial proportion of virally suppressed individuals and affecting executive function, attention, working memory, and processing speed [4]. Neuroimaging studies reveal cortical thinning, white matter abnormalities, and reduced brain volume in HAND patients, changes that parallel neurodegenerative diseases, including Alzheimer’s and Parkinson’s [5,6]. Critically, these structural changes reflect dendritic simplification, spine loss, and synaptic dysfunction rather than overt neuronal death, suggesting that functional impairment precedes cell loss and may be reversible with targeted intervention [7].

### 1.2. HIV-1 Entry into the Central Nervous System and Viral Protein Release

HIV-1 establishes CNS infection early in systemic infection, often before cART initiation, through the “Trojan horse” mechanism, in which infected monocytes and T-cells cross the blood–brain barrier (BBB) and deliver virus into the brain parenchyma [8,9,10]. Once established, HIV-1 infects perivascular macrophages, microglia, and astrocytes, which can release viral proteins under conditions of infection and immune activation, including gp120, Tat, Vpr, and Nef, as well as pro-inflammatory cytokines such as TNF-α, IL-1β, and IL-6, thereby contributing to neuroinflammatory and neurotoxic processes even under viral suppression [11,12]. In this regard, studies have reported detection of gp120 in cerebrospinal fluid (CSF) [13,14]. In addition, immunohistochemical studies have demonstrated gp120 localization in brain tissue, including perivascular macrophages and microglia in HIV-associated encephalitis [15]. Furthermore, experimental studies indicate that gp120 can cross the blood–brain barrier and enter the CNS, supporting its presence within the CNS [16]. Extracellular vesicles and exosomes serve as additional vehicles for dissemination, packaging viral proteins and pro-inflammatory cargo for cell-to-cell transfer in the absence of productive replication [17,18], potentially contributing to persistent neurocognitive impairment despite undetectable plasma viral loads. However, the precise quantitative levels of gp120 within CNS compartments remain incompletely defined and may vary with disease stage and sampling context.

### 1.3. gp120 as a Central Mediator of Neurotoxicity

Among the viral proteins released into the CNS, gp120 has emerged as a principal and mechanistically distinctive neurotoxic driver. gp120 is detected in cerebrospinal fluid at picomolar to nanomolar concentrations sufficient to trigger neuronal injury [13,19], and critically, these concentrations persist in virally suppressed individuals, establishing gp120 as a replication-independent source of ongoing CNS damage. Although neurons lack CD4 receptors and are not productively infected, they express the gp120 co-receptors CXCR4 and CCR5, enabling direct gp120 binding and downstream signal transduction [20,21]. gp120 gains intraneuronal access through lipid raft-mediated endocytosis, macropinocytosis, and dynein-mediated retrograde axonal transport following uptake at axon terminals [22], positioning it to act not only at the neuronal surface but within the cell body and along axons. Once internalized, gp120 has been proposed to broadly influence neuronal signaling networks and metabolic pathways, including calcium regulation, mitochondrial function, and neurotrophic signaling. It is important to note that gp120 acts within a complex inflammatory milieu in which multiple cytokines and chemokines also converge on overlapping signaling pathways. Gut-derived metabolites further amplify these effects by lowering the neuronal threshold for gp120-driven excitotoxic injury. This review examines each of these mechanisms in turn, building toward a unified framework that explains why gp120-driven neurotoxicity persists despite viral suppression and identifies the convergent therapeutic targets that a network-level disease demands [23,24,25,26].

## 2. GP120 Structure, Cellular Entry, and Distribution

### 2.1. gp120 Structure and Maturation

HIV-1 gp120 is a heavily glycosylated 120 kDa surface envelope protein encoded by the viral env gene. It is generated by furin-mediated cleavage of the gp160 precursor in the Golgi apparatus, yielding gp120 as the surface subunit and gp41 as the transmembrane subunit [27,28]. These subunits associate non-covalently to form trimeric spikes on the viral surface that mediate entry through CD4 and chemokine co-receptor binding. gp120 consists of five conserved regions (C1–C5) interspersed with five variable loops (V1–V5), with the V3 loop determining co-receptor tropism and the CD4-binding site residing within conserved regions [29]. Extensive N-linked glycosylation, with more than 24 glycans per monomer, shields immunogenic epitopes while also influencing co-receptor binding affinity and neurotoxic potency [30].

### 2.2. gp120 Binding to Neuronal Chemokine Receptors

Although neurons are not productively infected by HIV-1, they express the gp120 co-receptors CXCR4 and CCR5, particularly in the hippocampus, cortex, and basal ganglia—regions selectively vulnerable in HAND [31]. gp120 binding activates G protein-coupled receptor signaling cascades involving phospholipase C, protein kinase C, and mitogen-activated protein kinases [32]. CXCR4 engagement drives calcium influx, oxidative stress, and apoptosis, while CCR5 activation may paradoxically confer partial neuroprotection through Akt/PKB signaling [33]. Natural chemokine ligands, including SDF-1/CXCL12 for CXCR4 and RANTES/CCL5 for CCR5, competitively inhibit gp120 binding [34], and small-molecule CXCR4 antagonists such as AMD3100 attenuate gp120 neurotoxicity in vitro [35], establishing chemokine receptor engagement as a primary and pharmacologically tractable initiating event. Although CNS infection is predominantly associated with CCR5-tropic viral strains, both CCR5- and CXCR4-binding gp120 variants have been shown to activate overlapping downstream neurotoxic signaling pathways, supporting the use of either variant to interrogate conserved mechanisms of gp120-mediated neuronal injury.

### 2.3. gp120 Internalization and Intracellular Trafficking

Beyond surface receptor engagement, gp120 undergoes active internalization through several mechanisms that amplify and sustain its neurotoxic effects. gp120 co-localizes with cholesterol-rich lipid rafts and is internalized through clathrin-independent endocytosis; disruption of lipid rafts with methyl-β-cyclodextrin reduces both gp120 uptake and neurotoxicity [22,36,37]. In some cell types, CXCR4 activation additionally triggers macropinocytosis, enabling bulk gp120 internalization [38]. Following uptake at axon terminals, gp120 undergoes dynein-mediated retrograde transport to the cell body, a process associated with mitochondrial trafficking deficits and increased apoptosis [39]. gp120 is also packaged into exosomes and microvesicles released from infected cells, enabling cell-to-cell transfer that amplifies neurotoxic signaling and may protect gp120 from neutralization [40]. These internalization routes collectively extend gp120 neurotoxicity beyond the initial site of receptor engagement and contribute to its persistence in the CNS.

### 2.4. gp120 Fragmentation and Toxic Peptides

In vivo studies demonstrate that systemically injected gp120 is processed into smaller peptide fragments detectable by fast-performance liquid chromatography [41], although the functional consequences of these fragments in neuronal systems remain to be fully defined. These fragments may exhibit greater neurotoxicity than full-length gp120 through altered receptor interactions or enhanced membrane permeability, though their specific identities and mechanisms of action remain incompletely characterized and represent an important area for further investigation.

### 2.5. gp120 as a Vaccine Target and Implications for Neurotoxicity

gp120 was among the earliest HIV antigens pursued as a vaccine target, but gp120-based strategies failed to elicit broadly neutralizing immunity due to extensive glycan shielding, conformational masking of conserved epitopes, and pronounced sequence variability [42,43]. Structural analysis of gp120 in complex with CD4 demonstrates substantial conformational flexibility, including receptor-induced rearrangements that expose or occlude key epitopes, providing a mechanistic basis for immune evasion [44]. In parallel, dense glycosylation and ongoing antigenic variation further limit antibody recognition and clearance [45]. These same properties, structural flexibility, glycan shielding, and efficient shedding of gp120, are consistent with its persistence as a soluble, biologically active protein in the CNS despite effective antiretroviral therapy. Circulating and locally released gp120 retains signaling competence, sustaining domain-specific interactions with chemokine receptors and excitatory neurotransmitter systems independently of viral replication. While direct experimental evidence linking these structural features to CNS persistence remains limited, their established roles in immune evasion and antigen stability provide a plausible mechanistic basis for reduced clearance and prolonged neurotoxic activity. Thus, insights from gp120 structural biology and vaccine failure support a model in which the same molecular features that hinder neutralization may also contribute to sustained CNS dysfunction in HAND.

## 3. Direct Mechanisms of GP120-Induced Neurotoxicity

### 3.1. NMDA Receptor Dysregulation and Calcium Overload

#### 3.1.1. NMDA Receptor Structure and Function

N-methyl-D-aspartate receptors (NMDARs) are ionotropic glutamate receptors critical for synaptic plasticity, learning, and memory. Functional NMDARs are heterotetramers typically composed of two obligatory GluN1 subunits and two GluN2 (A–D) or GluN3 (A–B) subunits [46]. Activation requires both glutamate binding to GluN2 and glycine/D-serine binding to GluN1, as well as membrane depolarization to relieve Mg^2+^ block at the channel pore. Upon activation, NMDARs permit Ca^2+^ influx, initiating signaling cascades that underlie long-term potentiation (LTP) and memory formation [47]. However, excessive NMDAR activation, termed excitotoxicity, causes pathological Ca^2+^ overload, mitochondrial dysfunction, oxidative stress, and cell death [48]. NMDAR-mediated excitotoxicity is implicated in numerous neurodegenerative diseases, including Alzheimer’s, Huntington’s, and HAND [49].

#### 3.1.2. gp120-Induced NMDAR Hyperactivation

gp120 triggers NMDAR hyperactivation via multiple convergent mechanisms. It stimulates glutamate release from astrocytes and microglia while simultaneously reducing astrocytic glutamate uptake by downregulating excitatory amino acid transporter 2 (EAAT2/GLT-1), creating toxic synaptic glutamate accumulation [50]. gp120 also promotes surface trafficking and clustering of NMDARs on neuronal membranes through lipid raft stabilization and PKA/PKC-mediated phosphorylation of GluN1 subunits at serine 896/897, increasing synaptic NMDAR density [51]. gp120-induced IL-1β release from microglia drives Src-family kinase-mediated phosphorylation of GluN2B at tyrosine 1472, enhancing NMDAR activity and prolonging channel open time [52]. Finally, gp120 interferes with activity-dependent NMDAR internalization, preventing normal receptor downregulation and sustaining pathological activation [51]. Together, these mechanisms create a state of persistent NMDAR hyperactivation that drives the excitotoxic Ca^2+^ overload underlying gp120-induced neuronal injury.

#### 3.1.3. Calcium Dysregulation and Downstream Signaling

NMDAR hyperactivation drives excessive Ca^2+^ influx from the extracellular space. Additionally, gp120 stimulates Ca^2+^ release from endoplasmic reticulum stores via inositol 1,4,5-trisphosphate receptor (IP_3_R) and ryanodine receptor (RyR) activation [53]. This dual Ca^2+^ source overwhelms neuronal buffering capacity, leading to sustained cytosolic Ca^2+^ elevation that activates multiple Ca^2+^-dependent injury cascades. Ca^2+^-activated calpains degrade cytoskeletal, synaptic, and transcription proteins, contributing to dendritic spine loss and synaptic dysfunction [54]. Calcineurin, a Ca^2+^/calmodulin-dependent phosphatase, dephosphorylates and inactivates CREB, attenuating neuroprotective gene transcription as discussed in Section 5.1 [55]. Ca^2+^ activation of neuronal nitric oxide synthase generates nitric oxide that reacts with superoxide to form peroxynitrite, a potent oxidant that damages proteins, lipids, and DNA [56]. Excessive CaMKII activation, while essential for LTP at physiological levels, disrupts synaptic function and triggers apoptosis under the sustained Ca^2+^ elevations induced by gp120 [57].

#### 3.1.4. Pharmacological Blockade of NMDAR-Mediated Toxicity

NMDAR antagonists attenuate gp120 neurotoxicity in vitro and in vivo. Non-competitive antagonists, including MK-801 and memantine, block the channel pore, preventing pathological Ca^2+^ influx [58]. Memantine, clinically approved for Alzheimer’s disease, has been evaluated in limited pilot studies in HAND, with mixed outcomes, though complete NMDAR blockade impairs physiological synaptic plasticity. GluN2B-selective antagonists such as ifenprodil and Ro 25-6981 may offer a superior therapeutic window by preferentially targeting extrasynaptic NMDARs that mediate excitotoxicity while sparing synaptic NMDARs required for normal cognition [59]. The clinical and preclinical evidence for these and related strategies is reviewed in Section 9.

### 3.2. Voltage-Gated Potassium Channel Dysfunction

#### 3.2.1. Potassium Channels in Neuronal Excitability

Voltage-gated potassium (Kv) channels regulate neuronal excitability by controlling action potential repolarization and firing frequency. Kv2.1, a delayed-rectifier potassium channel abundantly expressed in hippocampal and cortical neurons, plays key roles in action potential repolarization and dendritic excitability [60].

#### 3.2.2. gp120-Induced Kv2.1 Dysregulation

gp120 binding to CXCR4 activates p38 MAPK, which phosphorylates Kv2.1 at serine 800, promoting channel insertion into the plasma membrane and increasing potassium efflux [61]. This hyperpolarizes neurons, reducing excitability and impairing action potential generation. Prolonged Kv2.1 hyperactivation triggers apoptosis through caspase-3 activation, likely by disrupting intracellular K^+^ homeostasis. Pharmacological Kv2.1 blockade with guangxitoxin-1E and genetic Kv2.1 knockdown both rescue neurons from gp120-induced apoptosis [62], as do CXCR4 antagonists and p38 MAPK inhibitors that prevent upstream Kv2.1 phosphorylation.

#### 3.2.3. Implications for Synaptic Transmission

Kv2.1 hyperactivation by gp120 reduces neuronal firing rates and impairs neurotransmitter release, contributing to synaptic dysfunction and cognitive impairment [63]. This mechanism may partially explain deficits in hippocampal LTP observed in gp120-exposed neurons and represents a pharmacologically tractable target, as discussed in Section 9.

### 3.3. Metabotropic Glutamate Receptors and Synaptic Plasticity

Metabotropic glutamate receptors (mGluRs) are GPCRs activated by glutamate that modulate synaptic transmission and plasticity. Group I mGluRs (mGluR1/5) enhance neuronal excitability and couple to Ca^2+^ mobilization, while Groups II (mGluR2/3) and III (mGluR4/6/7/8) suppress glutamate release and provide neuroprotection [64]. gp120 dysregulates mGluR signaling such that Group I mGluR overactivation exacerbates gp120-induced Ca^2+^ overload and oxidative stress, while Group II/III mGluR agonists confer neuroprotection in gp120-exposed neurons [65,66]. The precise mechanisms remain under investigation but likely involve altered mGluR trafficking, expression, or coupling to downstream effectors.

### 3.4. Interferon Signaling and the Ephrin-B/EphB Axis in gp120-Driven Neuroinflammation

gp120 induces type I interferon signaling in the CNS, which activates the ephrin-B/EphB axis as a mechanistically distinct neuroinflammatory pathway. Post-mortem HAND brain tissue shows elevated EphB2 that correlates with viral burden and inversely with cognitive performance [67]. Both gp120 and IFNβ drive upregulation of ephrin-B/EphB, and EphB2 activates microglia through reverse signaling via ephrin-B1, thereby promoting inflammatory factor secretion and contact-independent neurotoxicity; ephrin-B1 knockdown partially attenuates both effects [67]. The relationship between gp120 and IFNβ is not linear; IFNβ deficiency in gp120-transgenic mice worsens synaptic loss and memory impairment in a sex-dependent manner, indicating that endogenous IFNβ simultaneously sustains neuroinflammation and maintains neuronal homeostasis [68]. IFNβ knockout suppresses ERK1/2 and p38 MAPK activity in gp120-transgenic brains independently of sex, positioning MAPK signaling as a convergence point between interferon responses and the excitotoxic and mitochondrial cascades described in Section 3.1 and Section 4.

## 4. Mitochondrial Dysfunction and Bioenergetic Failure

### 4.1. Mitochondrial Structure and Function in Neurons

Mitochondria are double-membraned organelles that generate ATP via oxidative phosphorylation (OXPHOS), regulate Ca^2+^ homeostasis, govern apoptotic signaling, and produce reactive oxygen species (ROS) as metabolic byproducts. Neurons, with their high energy demands, extended morphologies, and limited glycolytic capacity, are exquisitely dependent on mitochondrial function [69]. Mitochondrial dynamics maintain mitochondrial health by enabling content mixing, distributing organelles along axons and dendrites, and removing damaged organelles via mitophagy [70]. Key proteins regulating fission include dynamin-related protein 1 (Drp1) and fission 1 (Fis1), while fusion is mediated by mitofusin 1/2 (Mfn1/2) and optic atrophy 1 (Opa1) [71]. Beyond these structural roles, emerging evidence indicates that gp120 induces metabolic reprogramming in neurons, shifting metabolism away from oxidative phosphorylation toward aerobic glycolysis, reducing ATP availability at synapses, and promoting pro-inflammatory transcriptional programs [72,73]. Dysregulation of glycolytic control nodes, including pyruvate kinase M2 (PKM2), may further couple this metabolic stress to altered calcium handling, oxidative stress, and CREB suppression [74], positioning metabolic reprogramming as a central amplifier of gp120-induced neurotoxicity rather than a secondary consequence of mitochondrial injury. Consistent with this, lipidomic profiling of gp120-transgenic mouse brains reveals elevated levels of inflammatory eicosanoids derived from the arachidonic acid cascade. Mechanistically, these changes are not limited to modulation of cyclooxygenase (COX) or lipoxygenase (5-LOX) expression; they instead reflect multilevel regulation of the pathway. In particular, LTC4 synthase (LTC4S), a downstream component of the 5-LOX branch, influences COX- and 5-LOX-related gene expression in a sex-dependent manner and modulates ERK1/2 and p38 MAPK signaling [75]. Given that MAPK activation can enhance cPLA2 activity and promote arachidonic acid release, these findings suggest that gp120 may amplify eicosanoid production through coordinated regulation of both upstream substrate availability and downstream enzymatic pathways, rather than through isolated control of COX or LOX enzymes alone. Together, these results identify arachidonic acid metabolism as a lipid-driven amplifier of gp120-induced neuroinflammation, intersecting with MAPK signaling and broader metabolic dysfunction.

### 4.2. gp120-Induced Mitochondrial Dysfunction

#### 4.2.1. Mitochondrial Calcium Overload

As described in Section 3.1.3, gp120 triggers cytosolic Ca^2+^ elevation via NMDAR activation and ER Ca^2+^ release. Mitochondria sequester excess cytosolic Ca^2+^ via the mitochondrial calcium uniporter (MCU) complex [66], but excessive uptake opens the mitochondrial permeability transition pore (mPTP), dissipating the mitochondrial membrane potential, halting ATP synthesis, and releasing pro-apoptotic factors [76]. Ca^2+^-stimulated OXPHOS simultaneously generates superoxide at Complexes I and III, damaging mitochondrial DNA, lipids, and proteins and impairing bioenergetic capacity [77]. Mitochondrial Ca^2+^ overload further compromises the organelle’s ability to buffer subsequent Ca^2+^ transients, exacerbating cytosolic Ca^2+^ dysregulation and creating a feed-forward cycle of excitotoxic and bioenergetic injury [78]. MCU inhibitors and genetic MCU deletion are neuroprotective in NMDAR excitotoxicity models and warrant evaluation in gp120-specific HAND contexts [79].

#### 4.2.2. Reactive Oxygen Species and Oxidative Stress

gp120 induces ROS production through multiple converging sources. Ca^2+^ overload and impaired electron transport chain function drive mitochondrial superoxide production, which is compounded by neuronal vulnerability due to relatively low SOD2 expression compared with astrocytes [80]. gp120 additionally activates microglial and neuronal NADPH oxidase enzymes, generating extracellular superoxide [81], while calcium-activated neuronal nitric oxide synthase produces nitric oxide that reacts with superoxide to form peroxynitrite, nitrating and inactivating proteins critical to neuronal function [82]. The cumulative oxidative burden damages mitochondrial DNA, gp120 increases the oxidative lesion 8-oxoG in neuronal mtDNA from HAND patients, impairing OXPHOS complex assembly and amplifying bioenergetic failure [83], while lipid peroxidation products, including 4-hydroxynonenal and malondialdehyde, compromise membrane integrity [84] and protein carbonylation disrupts enzyme function, cytoskeletal structure, and signal transduction [85]. Antioxidant strategies targeting these mechanisms are reviewed in Section 9.

#### 4.2.3. Impaired Mitochondrial Dynamics and Morphology

gp120 disrupts the balance of mitochondrial fission and fusion, driving fragmentation that impairs bioenergetic function [66]. gp120-induced Ca^2+^ overload and oxidative stress activate Drp1 through calcineurin-mediated dephosphorylation at Ser637 and CDK1/CDK5-mediated phosphorylation at Ser616, promoting Drp1 translocation to mitochondria and driving fission [66]. Concurrently, gp120 downregulates Mfn1, Mfn2, and Opa1, impairing fusion and preventing morphological recovery [86]. Electron microscopy reveals cristae disorganization and swelling in gp120-exposed neurons, reducing surface area available for OXPHOS complexes [26,86]. The resulting fragmented mitochondria exhibit reduced ATP production, increased ROS generation, impaired Ca^2+^ buffering, and disrupted axonal and dendritic trafficking, leaving synapses energy-depleted [26,66]. Pharmacological inhibition of Drp1 rescues mitochondrial morphology and attenuates gp120 neurotoxicity in preclinical models [87].

#### 4.2.4. Defective Mitochondrial Transport

Mitochondria undergo bidirectional transport along microtubules to meet spatially distributed energy demands in neurons, with anterograde transport mediated by kinesin motors and retrograde transport by dynein [88]. gp120 impairs this trafficking by interfering with kinesin-1 and dynein attachment to mitochondrial adaptor proteins Miro and TRAK/Milton [26], while elevated cytosolic Ca^2+^ causes Miro conformational changes that arrest mitochondrial movement and prevent redistribution to energy-depleted regions [89]. Oxidative stress and calpain activation further destabilize microtubules, compounding transport failure. The consequence is progressive energy deprivation in distal axons and dendrites, contributing to synaptic failure and neurite retraction that gp120-induced mitochondrial fragmentation initiates, and transport failure sustains.

#### 4.2.5. Defective Mitophagy

Mitophagy, the autophagic degradation of damaged mitochondria, is mediated by the PINK1-Parkin pathway: damaged mitochondria accumulate PINK1 on their outer membrane, recruiting Parkin, which ubiquitinates mitochondrial proteins and targets the organelle for degradation [90]. gp120 impairs this quality control pathway by disrupting PINK1 stabilization and Parkin translocation through oxidative and ER stress [86], reducing expression of autophagy-related proteins Atg5, Atg7, and Beclin-1 [91], and disrupting lysosomal acidification and protease activity, preventing autophagosome–lysosome fusion and cargo degradation [92]. Accumulation of damaged mitochondria compounds the bioenergetic failure, ROS production, and apoptotic signaling that gp120 drives through the mechanisms described above.

### 4.3. ER-Mitochondria Contact Sites (MAMs)

Mitochondria-associated ER membranes (MAMs) are specialized contact sites facilitating Ca^2+^ transfer, lipid synthesis, and apoptotic signaling between the ER and mitochondria [93]. MAMs are enriched in IP_3_Rs on the ER and voltage-dependent anion channels on mitochondria, enabling direct Ca^2+^ transfer. gp120 increases physical ER-mitochondria contacts, facilitating excessive Ca^2+^ transfer from IP_3_R-activated ER stores and exacerbating mitochondrial Ca^2+^ overload [94]. gp120 additionally modulates expression of MAM-resident proteins, including Mfn2, PACS2, and the sigma-1 receptor, disrupting lipid metabolism and increasing apoptotic susceptibility [95]. MAM dysregulation thus represents an upstream amplification point that integrates ER stress, mitochondrial Ca^2+^ overload, and apoptotic signaling in gp120 neurotoxicity.

### 4.4. Impaired Mitochondrial Biogenesis

Mitochondrial biogenesis is regulated by the transcriptional co-activator PGC-1α, which drives expression of nuclear-encoded mitochondrial genes, and by mitochondrial transcription factor A (TFAM), which regulates mtDNA replication. gp120 suppresses PGC-1α through three converging mechanisms: reduction in pCREB, which is a key transcriptional activator of PGC-1α as discussed in Section 5.1 [26]; downregulation of SIRT1, a deacetylase that activates PGC-1α by removing inhibitory acetyl groups [96]; and upregulation of miR-34a, which directly targets PGC-1α mRNA and reduces its translation [26]. The resulting impairment of mitochondrial biogenesis limits the cell’s capacity to replace damaged mitochondria, compounding the morphological, transport, and mitophagy deficits described above. Pharmacological strategies to restore PGC-1α and SIRT1 activity are reviewed in Section 9.

## 5. Transcriptional and Epigenetic Alterations

### 5.1. CREB Dysregulation

#### 5.1.1. CREB in Learning and Memory

cAMP response element-binding protein (CREB) is a transcription factor activated by phosphorylation at serine 133 (pCREB-Ser133), enabling it to recruit co-activators (CBP/p300) and drive gene transcription [97]. CREB is essential for long-term memory consolidation, synaptic plasticity, neuronal survival, and mitochondrial biogenesis [98]. Among its key transcriptional targets, CREB directly activates BDNF promoter IV, drives mitochondrial biogenesis through PGC-1α, promotes neuronal survival through Bcl-2 family members, and regulates immediate-early genes, including c-Fos, Arc, and Egr1, that are required for synaptic plasticity. CREB dysfunction is implicated in Alzheimer’s, Huntington’s, Parkinson’s, and depression [99], positioning it as a convergence point for neurodegenerative injury across multiple disease contexts.

#### 5.1.2. gp120-Induced CREB Inhibition

Multiple studies, including work from our laboratory, demonstrate that gp120 reduces CREB expression and pCREB levels in neurons through several converging mechanisms [26]. gp120-induced Ca^2+^ overload activates calcineurin, a phosphatase that dephosphorylates CREB at Ser133 and inactivates it [26]. gp120 also activates GSK-3β [100], which phosphorylates CREB at inhibitory sites [101], though whether this GSK-3β-mediated phosphorylation is a primary driver of CREB suppression downstream of gp120 has not been directly established. Reduced SIRT1 expression, a consequence of gp120-driven miRNA dysregulation discussed in Section 5.3, further impairs CREB deacetylation and activation [102]. Together, these mechanisms converge on sustained CREB suppression that reduces BDNF transcription, impairs mitochondrial biogenesis via PGC-1α, decreases Bcl-2-dependent survival signaling, and attenuates synaptic plasticity gene expression, resulting in cascading consequences that link gp120 receptor engagement to the full spectrum of synaptic and cognitive deficits observed in HAND.

### 5.2. BDNF Dysregulation

#### 5.2.1. BDNF in Neuronal Health and Plasticity

Brain-derived neurotrophic factor (BDNF) is a neurotrophin essential for neuronal survival, differentiation, synapse formation, and synaptic plasticity [103]. BDNF is synthesized as a pro-peptide (pro-BDNF, ~32 kDa), which is cleaved intracellularly by furin or extracellularly by plasmin to generate mature BDNF (mBDNF, ~14 kDa) [104]. Pro-BDNF and mBDNF have opposing functions that together determine the net neurotrophic or apoptotic outcome of BDNF signaling. mBDNF binds tropomyosin receptor kinase B (TrkB), activating PI3K/Akt, MAPK/ERK, and PLCγ pathways that promote survival, neurite outgrowth, and LTP [105]. Pro-BDNF, by contrast, binds the p75 neurotrophin receptor (p75NTR), activating JNK and caspase cascades that trigger apoptosis, long-term depression, and spine retraction [105]. The pro-BDNF/mBDNF ratio, therefore, determines whether BDNF signaling is neuroprotective or neurotoxic—a balance that gp120 directly disrupts.

#### 5.2.2. gp120 Disrupts BDNF Processinga and Therapeutic Implications

Mocchetti and colleagues demonstrated that gp120 increases pro-BDNF and reduces mBDNF in neurons and in HAND patient brains [106]. gp120 reduces furin and ADAM10 expression, impairing intracellular pro-BDNF cleavage [107], and downregulates tissue plasminogen activator (tPA), reducing extracellular pro-BDNF-to-mBDNF conversion [108]. gp120-induced CREB suppression further reduces BDNF promoter IV activity, limiting BDNF transcription at its source [72]. The resulting shift toward pro-BDNF drives neurite retraction via p75NTR/RhoA/ROCK-mediated cytoskeletal collapse, impairs synapse maintenance by disrupting mBDNF-TrkB signaling, promotes apoptosis via caspase activation, and undermines LTP by impairing CREB-dependent gene expression required for memory consolidation. This pro-BDNF accumulation, therefore, amplifies and sustains the synaptic injury initiated by gp120 through excitotoxic and mitochondrial mechanisms.

Exogenous mBDNF administration and TrkB agonists rescue neurons from gp120 toxicity in vitro and in vivo [109], but direct BDNF administration is limited by poor blood–brain barrier penetrance and short half-life. Small-molecule TrkB agonists, such as 7,8-dihydroxyflavone, show neuroprotective effects in HIV-1 transgenic models of HAND, where they improve mitochondrial dysfunction, synaptic integrity, and neuroinflammatory markers [110,111], supporting TrkB signaling as a potential neuroprotective strategy. Physical exercise increases hippocampal BDNF expression and neurogenesis in gp120-transgenic mice and improves cognitive function in people living with HIV, representing the only intervention with both preclinical mechanistic evidence and clinical support [112]. Ampakines, positive allosteric modulators of AMPA receptors that increase BDNF transcription, represent an additional strategy with preclinical rationale [113], though clinical evaluation in HAND remains limited.

### 5.3. MicroRNA Dysregulation

MicroRNAs (miRNAs) are ~22-nucleotide non-coding RNAs that post-transcriptionally regulate the expression of genes by binding complementary sequences in target mRNA 3’ untranslated regions, causing translational repression or mRNA degradation. gp120 directly alters neuronal miRNA expression profiles, and several of these changes have been functionally linked to synaptic dysfunction, mitochondrial failure, and inflammatory amplification in HAND.

#### 5.3.1. gp120-Regulated miRNAs with Direct Neuronal Targets

The most mechanistically characterized gp120-responsive miRNA is miR-34a, which is upregulated by gp120 through activation of p53 and NF-κB [26]. miR-34a directly targets PGC-1α and SIRT1 mRNA, suppressing mitochondrial biogenesis and CREB deacetylation, connecting gp120 receptor signaling directly to the bioenergetic and transcriptional deficits described in Section 4.4 and Section 5.1. miR-132, by contrast, is downregulated by gp120 despite being a CREB transcriptional target under physiological conditions. Loss of miR-132 impairs dendritic spine maintenance and reduces expression of synaptic proteins, including AMPA receptor subunits, contributing to the structural synaptic deficits observed in HAND [114]. The opposing dysregulation of these two miRNAs, one pathologically elevated, one pathologically depleted, illustrates how gp120 simultaneously drives injury and suppresses compensatory neuroprotective responses.

#### 5.3.2. Inflammatory miRNAs Amplify gp120-Driven Neuroinflammation

gp120 upregulates miR-155, a pro-inflammatory miRNA that enhances NF-κB signaling and suppresses negative regulators of cytokine production, thereby amplifying microglial and astrocytic inflammatory responses to gp120 receptor engagement [114]. Concurrently, miR-146a, which normally acts as a feedback suppressor of TLR and NF-κB signaling, is dysregulated by gp120, thereby impairing this anti-inflammatory brake [115]. Together, miR-155 upregulation and miR-146a dysregulation create a self-reinforcing inflammatory state that lowers the threshold for gp120-induced neuronal injury and sustains neuroinflammation beyond the duration of acute gp120 exposure.

#### 5.3.3. Limitations and Therapeutic Potential

A critical caveat is that several miRNA changes attributed to gp120 in the literature reflect indirect or multistep relationships; gp120 activates a signaling cascade that regulates a transcription factor that controls miRNA biogenesis, rather than direct gp120-miRNA coupling. Distinguishing primary gp120-driven miRNA changes from secondary downstream adaptations remains an important unresolved question. Therapeutically, antagomirs targeting miR-34a and miR-155 rescue neuronal function in gp120 models, while miR-132 mimics restore synaptic protein expression and dendritic complexity [114]. These proof-of-concept findings establish miRNA dysregulation as a pharmacologically tractable node in gp120 neurotoxicity, though CNS delivery of miRNA-targeting agents remains a significant translational barrier.

## 6. Cellular Senescence and Inflammaging

### 6.1. HIV-1 and Accelerated Aging

HIV-infected individuals exhibit premature aging phenotypes, including earlier onset of cardiovascular disease, osteoporosis, frailty, and neurocognitive decline. This “inflammaging”, chronic low-grade inflammation driving age-related pathology, is exacerbated by persistent immune activation despite viral suppression. gp120 directly contributes to this accelerated aging phenotype by inducing DNA damage, oxidative stress, and mitochondrial dysfunction in neurons and glia, driving cells prematurely into senescence through mechanisms that parallel but are distinct from those of replicative aging.

### 6.2. gp120-Induced Cellular Senescence

Cellular senescence is an irreversible growth arrest accompanied by secretion of pro-inflammatory cytokines, chemokines, and proteases, collectively termed the senescence-associated secretory phenotype (SASP). Recent evidence indicates that gp120 induces senescence in neurons, astrocytes, and microglia through genotoxic stress, oxidative damage, and sustained activation of inflammatory receptors [66]. Senescent cells in the gp120-exposed CNS exhibit upregulation of p16^INK4a^ and p21^CIP1^, senescence-associated beta-galactosidase activity, persistent DNA damage foci marked by gamma-H2AX and 53BP1, and robust SASP factor secretion, including IL-6, IL-8, TNF-alpha, MMP-3, and CCL2. SASP factors released by senescent glia amplify gp120 neurotoxicity by sustaining neuroinflammation and creating a feed-forward cycle of oxidative and inflammatory injury that persists independently of ongoing gp120 exposure [116].

### 6.3. Senescence Markers in HAND Patients

Post-mortem brain tissue and cerebrospinal fluid from HAND patients provide direct evidence that gp120-associated senescence is clinically relevant and not merely a cell culture phenomenon. Autopsy studies reveal elevated p16^INK4a^ expression in cortical neurons and astrocytes from individuals with HIV-associated dementia compared to age-matched HIV-negative controls and HIV-positive individuals without cognitive impairment [117]. p16^INK4a^-positive cells co-localize with markers of DNA damage and oxidative stress, consistent with gp120-induced genotoxic and oxidative insults driving premature senescence in vivo. CSF biomarker studies show that HIV-infected individuals with neurocognitive impairment have elevated SASP factors, IL-6, IL-8, MCP-1/CCL2, and MMP-9, that correlate inversely with neuropsychological test performance [118]. Critically, these markers remain elevated in virologically suppressed individuals on effective cART, indicating that gp120-driven cellular senescence persists independently of active viral replication and contributes to ongoing cognitive impairment. Peripheral senescent T cell burden, characterized by p16^INK4a^ and p21^CIP1^ expression, correlates with the severity of cognitive impairment, neurodegenerative biomarkers such as neurofilament light chain and tau, and neuroimaging measures of brain atrophy [119,120]. Telomere attrition is accelerated in HIV-infected brain tissue and associates with oxidative stress markers consistent with gp120-induced mitochondrial dysfunction [121]. HIV-infected astrocytes and microglia also exhibit telomere-independent stress-induced premature senescence, indicating that gp120 drives the senescent phenotype through multiple convergent mechanisms rather than solely through replicative exhaustion [121].

### 6.4. Therapeutic Targeting of Senescence

Senolytics selectively eliminate senescent cells, and senomorphics, which suppress SASP without inducing cell death, represent emerging therapeutic strategies with potential relevance to HAND. Dasatinib and quercetin, which target BCL-2 family anti-apoptotic pathways upregulated in senescent cells, reduce senescent cell burden, attenuate neuroinflammation, and improve synaptic density in gp120-transgenic mice [122,123]. A pilot clinical trial of dasatinib and quercetin in HIV-infected individuals demonstrated feasibility and preliminary reductions in systemic IL-6 and TNF-alpha, though neurocognitive endpoints were not assessed [123]. This evidence gap is important: preclinical senolytic data in HAND models are promising, but whether eliminating senescent cells translates to measurable cognitive benefit in HIV-infected individuals remains untested. Clinical trials currently underway in aging and Alzheimer’s disease will inform the design of HAND-specific trials, but direct evaluation of senolytic efficacy on neurocognitive outcomes in this population remains a priority.

## 7. Extracellular Vesicles and GP120 Dissemination

### 7.1. EVs as Mediators of gp120 Spread

Extracellular vesicles (EVs), including exosomes (30–150 nm) and microvesicles (100–1000 nm), are membrane-bound particles released by cells that transfer proteins, lipids, and nucleic acids to recipient cells [124]. In HIV neuropathogenesis, EVs released from infected macrophages, microglia, astrocytes, and brain endothelial cells propagate viral and inflammatory signals intercellularly. EVs can incorporate HIV-1 envelope glycoprotein gp120, enabling viral protein dissemination independent of productive viral replication [125,126]. Arakelyan et al. (2017) demonstrated that EVs from HIV-1-infected cells carry gp120 and enhance viral infection of human lymphoid tissue ex vivo, thereby establishing a functional role for Env-associated EVs in viral spread [40]. EV-associated gp120 may be partially shielded from antibody neutralization, prolonging its persistence in extracellular compartments and expanding its spatial range of activity within the CNS [126]. This mode of dissemination is particularly relevant in virologically suppressed individuals, where ongoing EV release may sustain gp120-mediated signaling despite undetectable plasma viremia.

### 7.2. Mechanisms of EV-Mediated Neurotoxicity

EV-associated gp120 is internalized by recipient neurons via endocytic and membrane fusion pathways, delivering the viral protein intracellularly independent of the classical receptor engagement required by soluble gp120 [126]. Once internalized, it activates downstream neurotoxic cascades, including calcium dysregulation, mitochondrial impairment, and oxidative stress. HIV-1 proteins gp120 and Tat also actively promote EV biogenesis in neurovascular compartments: endothelial and brain microvascular cells increase EV shedding upon exposure to these proteins, contributing to blood–brain barrier dysfunction [127,128]. EVs activate innate immune pattern-recognition receptors, including TLRs and cGAS-STING, in recipient cells, amplifying neuroinflammatory signaling [129], and EV-mediated delivery of inflammatory mediators and regulatory RNAs further increases neuronal susceptibility to excitotoxic and metabolic injury. Additionally, gp120-containing EVs facilitate CNS entry via transcytosis across brain endothelial cells, thereby bypassing the blood–brain barrier [130].

### 7.3. EV Biogenesis, Cellular Sources, and Key Neurotoxic Cargo

Multiple CNS-relevant cell types upregulate EV release under gp120 exposure. Endothelial cells exposed to gp120 or Tat increase microvesicle shedding and acquire a pro-inflammatory phenotype, releasing EVs that contain tight junction proteins such as occludin [127,128]. Macrophage-derived EVs transfer regulatory microRNAs that alter mitochondrial bioenergetics and epithelial barrier integrity [131]. These findings establish that gp120 is both a vesicle cargo and a stimulus that remodels EV biogenesis across vascular and immune compartments.

Beyond gp120 itself, EVs from HIV-infected cells carry additional cargo that compounds neuronal injury. EV-delivered Tat dysregulates transcription, while Nef disrupts endolysosomal trafficking and autophagy [132]. Pro-inflammatory cytokines (TNF-α, IL-1β, IL-6) and chemokines (MCP-1/CCL2, RANTES/CCL5) packaged within EVs amplify neuroinflammatory cascades in neurons that have not directly encountered free gp120 [133]. EV-mediated transfer of miR-34a, miR-29b, and miR-146a reprograms recipient neuron transcriptomes toward pro-apoptotic and anti-plasticity states by targeting CREB, PGC-1α, and inflammatory regulators [134]. EVs also deliver oxidized lipids, carbonylated proteins, and active NADPH oxidase subunits, which generate ROS in recipient cells, thereby compounding mitochondrial and oxidative injury initiated through receptor-mediated pathways [135]. EV-associated miR-23a and miR-27a further modulate mitochondrial function and metabolic pathways in recipient endothelial cells [131]. Collectively, these cargo components converge on the same excitotoxic, metabolic, and inflammatory nodes targeted by free gp120, positioning EVs as an amplification system rather than a parallel injury mechanism.

### 7.4. EV-Associated HIV Envelope in Particle Heterogeneity and Therapeutic Implications

HIV-1 gp120 can be incorporated into both virions and extracellular vesicles, generating heterogeneous mixed populations of infectious and non-replicative particles with overlapping physical properties. Arakelyan et al. (2017) showed that gp120-bearing EVs retain functional envelope activity sufficient to facilitate lymphoid tissue infection [40]. Troyer et al. (2024) demonstrated, using single-particle analysis, that viral RNA and gp120 coexist within heterogeneous nanoparticle populations, complicating the distinction between virions and EVs in biological samples [136]. EV-associated cargo also activates NF-κB signaling and inflammasome pathways in recipient cells, sustaining neuroinflammatory states [137]. Taken together, gp120 biological activity is distributed across a continuum of vesicle classes rather than restricted to free viral protein.

EV biogenesis and uptake pathways represent intervention points for limiting gp120-associated neurotoxicity. Neutral sphingomyelinase inhibitors such as GW4869 reduce EV biogenesis, decrease gp120 packaging into EVs, and limit dissemination to neurons [138]. Heparin and dynamin inhibitors block EV uptake in recipient cells, reducing intracellular gp120 delivery and its downstream excitotoxic and inflammatory consequences [138]. Targeting EV-associated microRNA transfer represents an additional strategy to mitigate mitochondrial dysfunction and inflammatory reprogramming [131]. Engineered EVs loaded with neuroprotective cargo, mBDNF, antioxidants, or miRNA mimics targeting miR-34a and miR-155, represent an emerging delivery strategy that exploits the same cellular uptake machinery to deliver therapeutic rather than toxic payload. Clinical translation remains early, and specificity, brain penetrance, and safety of EV-modulating agents in humans require further investigation before application in HAND.

## 8. Gut–Brain Axis as a Systemic Amplifier of GP120 Neurotoxicity

### 8.1. HIV-Induced Gut Barrier Dysfunction Primes CNS Vulnerability

HIV-1 infection depletes CD4^+^ T cells in gut-associated lymphoid tissue (GALT), disrupting the integrity of the intestinal epithelial barrier and enabling microbial translocation even with effective cART [139]. Circulating bacterial products, particularly lipopolysaccharide (LPS), activate peripheral immune cells and drive sustained production of TNF-α, IL-1β, and IL-6, which then enter the CNS through circumventricular organs and compromised segments of the blood–brain barrier [140]. This systemic inflammatory milieu primes microglia and astrocytes toward a hyperreactive phenotype—critically, one in which subsequent gp120 exposure triggers disproportionately amplified glutamate release, cytokine production, and ROS generation compared to gp120 alone. The gut–brain axis, therefore, does not initiate gp120 neurotoxicity independently but lowers the threshold at which gp120 drives excitotoxic and oxidative injury [141].

### 8.2. Quinolinic Acid Is the Principal Gut-Derived Synergist of gp120 Excitotoxicity

Among microbiome-derived metabolites, the kynurenine pathway product, quinolinic acid, represents the most direct molecular link between gut dysbiosis and gp120 neurotoxicity [141]. HIV infection shifts tryptophan metabolism toward quinolinic acid production by enhancing host indoleamine 2,3-dioxygenase (IDO) activity, while gut dysbiosis simultaneously reduces bacterial tryptophan conversion to anti-inflammatory indole derivatives [142]. The resulting accumulation of quinolinic acid in CSF synergizes directly with gp120-driven NMDAR hyperactivation: both act on the same receptor population, causing additive Ca^2+^ overload, mitochondrial dysfunction, and excitotoxic neuronal injury that neither stimulus alone achieves. Quinolinic acid further impairs astrocytic glutamate uptake, reducing the buffering capacity that normally limits gp120-induced excitotoxicity. This convergence at the NMDAR makes the kynurenine pathway a tractable therapeutic target. IDO inhibitors reduce the quinolinic acid burden, while NMDAR antagonists such as memantine address the downstream excitotoxic consequences of both gp120 and quinolinic acid [143].

### 8.3. SCFA Deficiency Shifts Microglia Toward a gp120-Hypersensitive State

Gut microbiome dysbiosis in HIV infection depletes the short-chain fatty acid (SCFA)-producing bacteria, reducing butyrate and propionate levels [144]. These SCFAs normally act as HDAC inhibitors in microglia, suppressing NF-κB-driven pro-inflammatory transcription and maintaining phagocytic and anti-inflammatory functions. SCFA deficiency shifts microglia toward a glycolytic, inflammasome-competent state that overresponds to gp120 receptor engagement, amplifying IL-1β and TNF-α secretion and impairing clearance of gp120-damaged cellular debris [145]. Restoring SCFA levels through prebiotic supplementation or probiotic reconstitution of SCFA-producing species attenuates microglial hyperreactivity and may reduce the neuroinflammatory amplification of gp120 toxicity, though direct clinical evidence in HAND remains limited.

### 8.4. Therapeutic Implications

Given that the gut–brain axis primarily functions as an amplifier rather than an independent driver of gp120 neurotoxicity, microbiome-targeted interventions are unlikely to be sufficient on their own. Their greatest clinical utility will be as adjuncts—reducing the systemic inflammatory baseline against which gp120 operates. Combinations of IDO inhibitors or NMDAR modulators with microbiome-restoration strategies represent a rational approach to disrupting the quinolinic acid-gp120 excitotoxic axis, while probiotic or prebiotic interventions that target SCFA restoration may reduce microglial sensitization. Clinical trials integrating these strategies with direct neuroprotectants are warranted.

## 9. Therapeutic Strategies and Future Directions

### 9.1. Limitations of Current Antiretroviral Therapy

Combination antiretroviral therapy effectively suppresses systemic viral replication but does not fully prevent HAND. Several factors explain this limitation. Many antiretrovirals exhibit incomplete penetration of the blood–brain barrier, allowing viral persistence within CNS reservoirs [146]. Chronic immune activation and neuroinflammation persist despite systemic viral suppression, and certain antiretrovirals, including efavirenz, may exert direct neurotoxic effects [147]. Critically, latently infected cells continue to release gp120 during effective viral suppression, thereby sustaining excitotoxic, inflammatory, and mitochondrial stress pathways in the CNS independently of replication [13]. These limitations underscore the need for adjunctive neuroprotective strategies that directly target gp120-driven mechanisms.

### 9.2. Clinical and Preclinical Evidence for Targeted Interventions

The mechanistic pathways described in this review converge on a limited number of pharmacologically tractable nodes, and therapeutic evidence varies considerably across them (Table 1).

NMDAR modulation has one of the more developed translational foundations, but remains inconclusive in HAND. Memantine, a low-affinity NMDAR antagonist approved for Alzheimer’s disease, has been tested in small clinical studies in HAND with variable results [148]. More selective inhibition of extrasynaptic GluN2B-containing NMDARs may improve the therapeutic window by suppressing excitotoxic signaling while preserving synaptic NMDAR function required for normal cognition [149]. Combining memantine with IDO inhibitors simultaneously reduces the quinolinic acid burden and addresses gp120-driven and gut-derived NMDAR hyperactivation through complementary mechanisms.

Mitochondrial protection and antioxidant strategies are supported by robust preclinical evidence but limited clinical data. N-acetylcysteine replenishes glutathione and reduces HIV-associated oxidative stress, while mitochondria-targeted antioxidants such as MitoQ directly scavenge ROS at their sites of generation [150,151]. NAD^+^ precursors, including nicotinamide riboside, improve mitochondrial metabolism and stress resistance in preclinical models, and PGC-1α activators, such as bezafibrate and resveratrol, might thus enhance mitochondrial biogenesis downstream of gp120-induced CREB suppression [152]. None of these agents has been evaluated in adequately powered HAND-specific clinical trials, representing a significant translational gap.

**Table 1:** Therapeutic strategies targeting HIV-1 gp120-mediated neurotoxicity. Therapeutic approaches are organized by primary molecular target and include both single-agent and combination strategies. The table summarizes the mechanism of action, key preclinical evidence demonstrating efficacy against gp120 neurotoxicity, current clinical development status in HIV-associated neurocognitive disorders (HAND) or related conditions, and representative references. Preclinical evidence includes in vitro neuronal culture studies, ex vivo brain slice experiments, and in vivo rodent/primate models demonstrating neuroprotection against gp120-induced injury. Clinical status indicates the most advanced stage of human testing for HAND, specifically, though some agents are approved for other indications. NMDAR modulators target excitotoxicity and calcium dysregulation. Mitochondrial therapies address bioenergetic failure, ROS production, and organellar dysfunction. BDNF/neurotrophin strategies restore neurotrophic support and synaptic plasticity. Chemokine receptor antagonists block gp120 binding and signaling. Anti-inflammatory agents suppress glial activation and cytokine production. Senolytics eliminate senescent cells that amplify neurotoxicity via SASP. EV inhibitors prevent intercellular transfer of toxic cargo. Gut–brain axis modulators reduce systemic inflammation and restore CNS homeostasis. Given the multifactorial nature of gp120 neurotoxicity, involving converging pathways of excitotoxicity, mitochondrial dysfunction, oxidative stress, inflammation, and senescence, combination therapies targeting multiple mechanisms simultaneously may offer superior efficacy compared to single-agent approaches. Several combinations are proposed based on mechanistic rationale but await formal clinical evaluation.

Therapeutic Target	Agent/ Intervention	Mechanism of Action	Preclinical Evidence	Clinical Status	Refs.
NMDA Receptors	Memantine	Low-affinity uncompetitive NMDAR antagonist	Reduces gp120 toxicity in neurons; improves cognition endpoints in preclinical models	Limited clinical studies in HAND; mixed results	[58,149]
	Ifenprodil	Selective GluN2B antagonist; blocks extrasynaptic neurotoxic signaling	Prevents gp120-induced calcium overload and apoptosis	Preclinical only	[59,150]
Calcium Signaling	MCU inhibitors (Ru360)	Block mitochondrial calcium uniporter	Reduces gp120-induced mitochondrial dysfunction and ROS	Preclinical; safety concerns for systemic use	[79]
	Nimodipine	L-type voltage-gated Ca^2+^ channel blocker	Attenuates gp120-induced Ca^2+^ influx and neurotoxicity	Preclinical; used clinically for stroke but not tested in HAND	—
Mitochondria	MitoQ	Mitochondria-targeted ubiquinone antioxidant	Reduces gp120-induced ROS; improves mitochondrial function	Preclinical; Phase II trials in other diseases	[151]
	Elamipretide (SS-31)	Mitochondria-targeted peptide; stabilizes cardiolipin	Enhances mitochondrial function; reduces oxidative stress	Preclinical in HAND; Phase II trials in heart failure	—
	PGC-1α activators (bezafibrate, ZLN005)	Induce mitochondrial biogenesis via PGC-1α upregulation	Restores mitochondrial number and function in gp120 models	Preclinical; bezafibrate FDA-approved for dyslipidemia	[72,73]
	Nicotinamide riboside/NMN	NAD^+^ precursors; activate sirtuins, enhance mitochondrial function	Improves bioenergetics; reduces gp120-induced dysfunction	Clinical trials in aging; preliminary data in HIV	[153]
Oxidative Stress	N-Acetylcysteine (NAC)	Glutathione precursor; replenishes antioxidant defenses	Reduces gp120-induced ROS and neurotoxicity	Phase II trials in HIV show improved immune function	[85]
	α-Lipoic acid	Mitochondrial antioxidant; metal chelator	Protects against gp120-induced oxidative damage	Preclinical; used clinically for diabetic neuropathy	—
BDNF/Neurotrophins	7,8-Dihydroxyflavone	Small-molecule TrkB agonist; mimics BDNF signaling	Rescues neurons from gp120 toxicity; promotes synaptic plasticity	Preclinical; Phase I safety trials completed	[105,109,110,111]
	LM22A-4	Selective TrkB partial agonist	Promotes neuronal survival; improves cognition in animal models	Preclinical; in development	[105]
	Exercise	Increases endogenous BDNF expression; enhances neurogenesis	Improves hippocampal function in gp120-transgenic mice	Recommended for HIV patients; ongoing trials	[112,154]
Chemokine Receptors	Maraviroc	CCR5 antagonist; FDA-approved antiretroviral	Blocks R5-tropic gp120 binding; anti-inflammatory effects	Phase II trials in HAND; mixed neurocognitive results	[33,35]
	AMD3100 (Plerixafor)	CXCR4 antagonist; FDA-approved for stem cell mobilization	Blocks X4-tropic gp120 binding; reduces neurotoxicity in vitro	Preclinical for HAND; limited CNS penetration	[35]
Inflammation	Minocycline	Tetracycline antibiotic; inhibits microglial activation	Reduces gp120-induced neuroinflammation and synaptic damage	Phase II trials in HAND; some cognitive benefit	[155]
	Anti-TNF-α (etanercept)	TNF receptor fusion protein; neutralizes TNF-α	Reduces neuroinflammation in animal models	Preclinical; safety concerns in HIV	—
Senescence	Dasatinib + Quercetin	Senolytic combination; targets BCL-2 family proteins	Clears senescent glia; reduces SASP and neuroinflammation	Pilot trial in HIV shows reduced systemic inflammation; neurocognitive outcomes pending	[123,124]
	Fisetin	Natural senolytic flavonoid	Eliminates senescent cells; improves cognition in aged mice	Preclinical in HAND models; Phase II trials in aging	[123]
Extracellular Vesicles	GW4869	Neutral sphingomyelinase inhibitor; blocks EV release	Reduces EV-mediated gp120 transfer and neurotoxicity	Preclinical only	[133]
	Heparin	Inhibits EV uptake; blocks cell surface binding	Prevents EV-mediated delivery of toxic cargo	Preclinical; clinical use for other indications	[124]
Gut–Brain Axis	Probiotics	Restore gut microbiome; reduce microbial translocation	Decreases LPS, systemic inflammation, improves gut barrier	RCTs in HIV show reduced inflammation; neurocognitive outcomes under study	[140,142]
	Prebiotic fibers	Enhance SCFA production; support beneficial bacteria	Improves BBB integrity; reduces microglial activation	Pilot studies in HIV show improved gut barrier function	[145]
	FMT	Transfers healthy donor microbiome	Restores gut ecology; reduces inflammation	Pilot studies in SIV models show feasibility; human trials planned	[140]
Combination Strategies	cART + memantine + NAC	Viral suppression + NMDAR blockade + antioxidant	Addresses multiple convergent pathways synergistically	Conceptual; no formal trials of this specific combination	[147,148]
	cART + D + Q + exercise	Viral suppression + senolytic + BDNF enhancement	Targets inflammation, senescence, and neuroplasticity	Under investigation in aging HIV populations	[122]

Abbreviations: NMDAR, N-methyl-D-aspartate receptor; MCU, mitochondrial calcium uniporter; ROS, reactive oxygen species; oxidative phosphorylation; PGC-1α, peroxisome proliferator-activated receptor gamma coactivator 1-alpha; NAD^+^, nicotinamide adenine dinucleotide; NMN, nicotinamide mononucleotide; BDNF, brain-derived neurotrophic factor; TrkB, tropomyosin receptor kinase B; TNF, tumor necrosis factor; SASP, senescence-associated secretory phenotype; EV, extracellular vesicle; SCFA, short-chain fatty acid; BBB, blood–brain barrier; FMT, fecal microbiota transplantation; cART, combination antiretroviral therapy; RCT, randomized controlled trial; SIV, simian immunodeficiency virus.

Neurotrophic support addresses the CREB-BDNF suppression that gp120 drives through calcineurin activation and miRNA dysregulation. Physical exercise increases hippocampal BDNF and improves cognitive function in HIV-infected individuals, representing a non-pharmacological intervention with clinical evidence [153]. Direct BDNF administration remains limited by poor BBB permeability and a short half-life, making small-molecule TrkB agonists a more viable clinical strategy.

Anti-inflammatory and senolytic approaches target the neuroinflammatory amplification of gp120 toxicity. Minocycline showed mixed results in HAND trials [154], whereas TNF-alpha inhibitors reduce neuroinflammation in experimental models, although there is no established clinical evidence for HAND. Dasatinib and quercetin have been shown to reduce senescent cell burden and SASP-driven neuroinflammation in transgenic mice [155]; therefore, it is worth testing this combination in gp120-tg mice.

Chemokine receptor antagonists offer a direct strategy for reducing gp120 receptor engagement in the CNS. Maraviroc, a CCR5 antagonist used clinically as an antiretroviral, demonstrates anti-inflammatory and neuroprotective effects beyond viral suppression. Experimental studies show that CXCR4 antagonists, including AMD3100, block gp120 binding and reduce neurotoxicity, although CNS pharmacokinetics and clinical application require further development.

### 9.3. The Case for Combination Therapy

The mechanistic framework presented in this review makes clear that no single-target intervention will be sufficient. gp120 drives neurotoxicity through simultaneously excitotoxic, metabolic, and inflammatory axes, and these pathways reinforce one another. NMDAR-driven Ca^2+^ overload induces mitochondrial dysfunction, suppresses CREB and BDNF, and reduces resilience to further excitotoxic insults. Interrupting one node without addressing the others leaves self-reinforcing injury cycles intact. Rational combination strategies should therefore target convergence points rather than individual pathways: pairing NMDAR modulation with mitochondrial antioxidants addresses both the initiating Ca^2+^ overload and its bioenergetic consequences; adding neurotrophic support restores the CREB-BDNF axis that gp120 suppresses; and incorporating anti-inflammatory or senolytic agents disrupts the SASP-driven amplification that sustains neuroinflammation beyond acute gp120 exposure. Microbiome-targeted interventions are best positioned as adjuncts that reduce the systemic inflammatory baseline rather than as primary neuroprotective agents.

### 9.4. Priorities for Clinical Translation

Several barriers must be addressed before mechanism-based HAND therapies reach clinical application. Validated CNS biomarkers, EV-associated gp120, CSF SASP factors, mitochondrial stress markers, and synaptic integrity measures are needed to identify individuals at risk, stratify treatment, and monitor therapeutic response. Precision medicine approaches that incorporate genetic risk factors, including APOE4 and sex as biological variables, will be essential, given documented differences in gp120 sensitivity across these strata. Improved experimental platforms, including human brain organoids and humanized mouse models, are enabling more physiologically relevant testing of gp120 therapeutics. Adequately powered longitudinal clinical trials integrating neurocognitive assessment, neuroimaging, and biomarker endpoints are ultimately required to determine whether targeting gp120-driven mechanisms produces meaningful and durable cognitive benefit in HAND.

## 10. Conclusions

HIV-1 gp120 emerges as a convergent driver of neurocognitive decline, integrating excitotoxic signaling, mitochondrial failure, and chronic inflammation into a self-reinforcing network that destabilizes synapses and accelerates brain aging. Rather than acting through isolated pathways, gp120 functions as a systems-level reprogrammer, linking Ca^2+^ dysregulation to metabolic collapse and suppressing CREB-BDNF-dependent resilience, while senescence-associated inflammatory secretion and extracellular vesicle-mediated signals amplify and propagate injury across the CNS. Gut-derived metabolites, particularly quinolinic acid, further lower the threshold for excitotoxic injury by converging on the same NMDAR populations that gp120 hyperactivates, positioning the gut–brain axis as a systemic amplifier rather than an independent pathogenic mechanism. This integrated framework explains a central clinical paradox: neurocognitive impairment persists despite effective viral suppression because gp120-driven toxicity operates independently of active replication. Progress will therefore require shifting from single-target interventions to mechanism-based combination strategies that simultaneously disrupt convergent nodes of excitotoxicity, mitochondrial dysfunction, and neuroinflammation while restoring neurotrophic capacity. In this context, HAND is best understood not as a residual complication of infection but as a network disease with identifiable and therapeutically actionable points of convergence.

## 11. Knowledge Gaps and Future Directions

**Figure 1:**
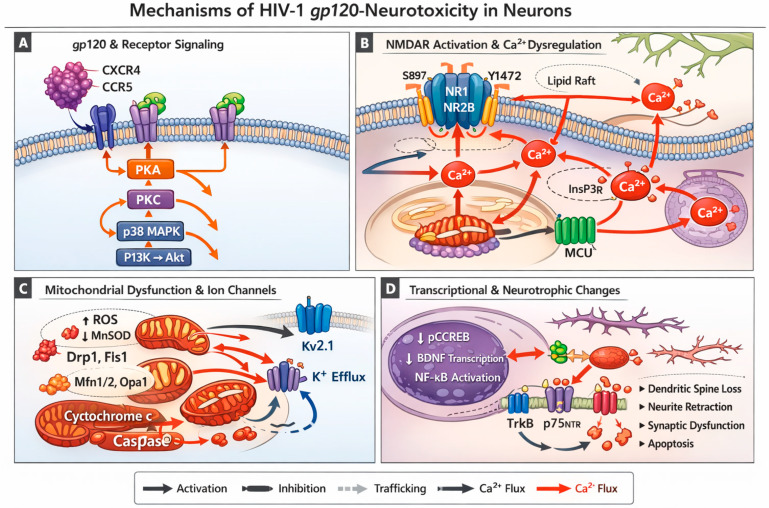
Mechanisms of HIV-1 gp120–induced neuronal injury. This schematic summarizes the signaling and downstream pathological effects of HIV-1 gp120 in neurons, integrating receptor activation, excitotoxic signaling, mitochondrial dysfunction, and transcriptional regulation. (**A**) gp120 and receptor signaling. HIV-1 gp120 engages neuronal surface receptors, including chemokine receptors and neurotrophic-associated receptors, initiating early intracellular signaling cascades. (**B**) NMDAR activation and Ca^2+^ dysregulation. gp120 enhances NMDAR-mediated signaling, leading to excessive Ca^2+^ influx and disruption of intracellular calcium homeostasis. Ca^2+^ flux is depicted by red arrows throughout this panel, illustrating entry through NMDARs and subsequent redistribution to intracellular compartments, where it serves as a central driver of downstream neuronal injury pathways. (**C**) Mitochondrial dysfunction and ion channel dysregulation. Elevated intracellular Ca^2+^ contributes to mitochondrial impairment, ROS production, and altered ion channel function, collectively disrupting neuronal bioenergetics and excitability. Inhibitory interactions are indicated by blunt-ended lines, as shown for regulatory signaling steps affecting mitochondrial and ion channel pathways. (**D**) Transcriptional and neurotrophic dysregulation. gp120-associated signaling alters transcriptional programs and disrupts neurotrophic receptor balance (including TrkB and p75NTR), shifting neuronal responses away from survival and toward injury-related pathways. Inhibitory signaling is indicated by blunt-ended lines within this panel. Legend key. Arrows indicate activation or stimulation; red arrows indicate Ca^2+^ flux; blunt-ended lines indicate inhibition; dashed arrows indicate trafficking or indirect effects.

Despite substantial progress in defining the mechanisms of gp120 neuronal impact (Figure 1), critical gaps remain that limit the translation of these mechanisms into effective therapies. The question of reversibility is perhaps the most clinically urgent; it remains unknown whether gp120-induced synaptic loss, mitochondrial dysfunction, and senescent cell accumulation can be meaningfully rescued once established, or whether intervention must occur early to prevent irreversible structural damage. Closely related is the challenge of distinguishing causality from compensation: the signaling changes documented in gp120-exposed neurons reflect a mixture of primary injury mechanisms and adaptive responses, and current experimental approaches cannot reliably distinguish between them. Without this distinction, therapeutic targets identified in vitro may reflect compensatory pathways whose inhibition worsens rather than improves outcomes. The coupling between metabolic reprogramming and neuroinflammation also remains poorly resolved. It is unclear how the glycolytic shift that gp120 drives in neurons and microglia evolves over time, whether it precedes or follows inflammatory activation, and whether it is reversible with metabolic interventions. In the EV field, the specific cargo components causally responsible for neurotoxicity have not been separated from bystander molecules, a distinction essential for developing EV-targeted therapeutics with acceptable selectivity. At the circuit level, the basis for selective vulnerability of specific neuronal populations—hippocampal, frontostriatal, and cerebellar—to gp120-driven injury remains mechanistically unexplained, limiting the development of region-specific interventions. Finally, whether the convergent pathway targeting strategies proposed in this review can restore cognitive resilience in older individuals with established HIV infection, rather than merely slowing further decline, is an open question that only adequately powered longitudinal clinical trials can resolve. Addressing these gaps will require experimental platforms that more closely recapitulate the chronic, low-level gp120 exposure observed in virologically suppressed HAND, including human brain organoids, humanized mouse models with aging components, and post-mortem tissue studies with detailed clinical correlation.

## Data Availability

No new data were created or analyzed in this study.
